# Supplementary material for: A study of competitions in different fields through graphs under bipolar picture fuzzy environment
Source: PLoS One. 2025 Oct 10;20(10):e0329192. doi: 10.1371/journal.pone.0329192 (PMC12513671; doi:10.1371/journal.pone.0329192)
Supplement: Attachment — Submitted filename: Reviwers comments with Responses.docx [file pone.0329192.s001.docx]

**Reviewer-1 Comments with Responses:**

**Manuscript ID:** PONE-D-25-05783

**Title:** A study of competitions in different fields through graphs under bipolar picture fuzzy environment

We appreciate the reviewer for their time and precious comments. No doubt, after endorsing these comments our article would become more effective. The point by point response of the reviewers comments are as follows.

**Reviewer #1:**

The paper introduces the concept of bipolar picture fuzzy competition graphs (BPFCGs) as an extension of fuzzy competition graphs. It claims to enhance the modeling of real-world competitive systems through a more comprehensive representation of uncertainty. The authors provide mathematical formulations and comparative analyses to support their argument.

While the study contributes to the field of fuzzy graph theory, its practical implications remain ambiguous. The paper lacks explicit real-world case studies that illustrate the necessity of this extended model over traditional competition graphs. Providing concrete applications in business, economics, or ecological modeling would strengthen the paper's impact. To enhance the quality and impact of the paper, the authors should consider the following:

Here are 20 suggestions for improving the paper:

**Comment#1:** Make the abstract more concise and the introduction more engaging by highlighting key contributions and applications.

**Response.** In accordance with the reviewer's suggestion, we have revised the abstract to enhance clarity and conciseness. Furthermore, we have also highlighted key contributions in the introduction section.

**Comment#2:** Reduce redundancy and simplify mathematical expressions to improve readability, especially for those unfamiliar with fuzzy graph theory.

1. Eliminate Redundant Explanations, like

A fuzzy set assigns a degree of membership to each element. This degree of membership is a number between 0 and 1.” Simplify to: “A fuzzy set assigns a membership value in [0, 1] to each element.”

2. Use Simpler Notation and Language, Add Brief Intuitive Descriptions

After formal definitions, add a simple sentence for readers unfamiliar with fuzzy theory.

Example: “The fuzzy degree reflects how strongly a node or connection is present in the system.”

3. Streamline Complex Expressions

Break long equations or conditions into smaller, readable components.

Use bullets or numbered steps for algorithmic parts or long logical statements.

**Response.** As per reviewer’s suggestions, we have removed redundancies wherever found, added sentences before definitions, further elaborated the terms through geometrical representations. Adjusted long equations and equation numbers are also allocated.
**Comment#3:** Provide specific real-world case studies to demonstrate how BPFCGs improve competition modeling in business, economics, or ecology.

**Response.** We have added specific real-world case studies to demonstrate how BPFCGs improve competition modeling in section 6: Real-World Application: Modeling Product

Competition Using BPFCGs. Moreover, in sections 4.1, 4.2, 4.4, we have also highlighted that how BPFCGs improve competition modeling in business, economics, or ecology.
**Comment#4:** Clearly differentiate BPFCGs from prior models in terms of computational efficiency, predictive accuracy, or applicability.

**Response.** Thank you for the insightful comment. We have expanded our discussion to clearly highlight the advantages of **Bipolar Picture Fuzzy Competition Graphs (BPFCGs)** over traditional **Picture Fuzzy Competition Graphs (PFCGs)** through Tables 20 & 21.

**Comment#5:** Conduct experiments using real-world datasets instead of relying solely on theoretical justifications.

**Response.** We have added specific real-world case studies to demonstrate how BPFCGs improve competition modeling in section 6: Real-World Application: Modeling Product

Competition Using BPFCGs.
**Comment#6:** Compare BPFCGs with existing competition graph models using concrete measures such as accuracy, computational complexity, and scalability.

**Response.** We have compared BPFCGs with existing competition graph models using concrete measures such as accuracy, computational complexity, and scalability in each subsection of the section 4. We constructed several tables in the section 5: Comparative study and prevalence of our presented model.
**Comment#7:** Describe in detail the computational methods used to generate BPFCGs and explain their efficiency.

**Response.** We have described the computational methods used to generate BPFCGs after defining BPFCGs, and explain their efficiency in detail in different sections like section 3 (theoretical aspect)*,* section 4 (general application point of view) and section 6 (real-word data perspective).
**Comment#8:** Compare BPFCGs with a broader range of fuzzy and competition graph models to justify their necessity.

**Response.** We have compared BPFCGs with a broader range of fuzzy and competition graph models to justify their necessity in Section 5.1, “Prevalence of our presented model for mobile companies through BPFCGs (Tables 19, 20 & 21)*.”*

**Comment#9:** Discuss the constraints and weaknesses of BPFCGs, such as computational overhead or specific application constraints.

**Response.** We have highlighted the constraints and weaknesses of BPFCGs, such as computational overhead or specific application constraints in section 7.

**Comment#10:** Remove redundant definitions and explanations that do not contribute to new insights.

**Response.** As per reviewer’s suggestion, we have removed redundant definitions and explanations that do not contribute to new insights.

**Comment#11:** Streamline the introduction, definitions, and results sections to enhance logical flow.

**Response.** As per reviewer’s suggestion, we have streamlined introduction, definitions, and results sections to enhance logical flow.

**Comment#12:** Explain the real-world significance of each theorem and derivation to make the findings more accessible.

**Response.** We thank the reviewer for their thoughtful suggestion. We respectfully note that the manuscript already includes detailed applications that illustrate the real-world relevance of the theoretical framework of BPFCGs. These applications are directly supported by the presented theorems and derivations. Nonetheless, we have reviewed the manuscript carefully and added brief clarifying remarks where needed to further emphasize the real-world significance of key results.

**Comment#13:** Provide a deeper discussion on the results, including how BPFCGs can be integrated into various research areas.

**Response.** We have added more information and also highlighted how BPFCGs can be integrated into various research areas in Section 6, “Conclusion”.
**Comment#14:** Use visual representations, graphs, or real-world examples to clarify complex concepts.

**Response.** We have added more visual representations in section 2: Preliminaries, and also highlighted graphs and real-world examples to clarify complex concepts in the whole article.
**Comment#15:** Ensure that all terms and abbreviations are consistently used throughout the paper.

**Response.** As per the reviewer’s suggestion, we have revised and checked that all terms and abbreviations are consistently used throughout the paper.

**Comment#16:** Provide a clear step-by-step explanation of how BPFCGs are constructed and analyzed.

**Response.** We have provided and highlighted a clear step-by-step explanation of how BPFCGs are constructed and analyzed in Section 3, Example 3.1. Moreover, in application section, we have also elaborated this point.

**Comment#17:** Suggest specific future work, such as algorithm optimization, application in new domains, or improvements in computational efficiency.

**Response.** We have provided and highlighted specific future work, such as algorithm optimization, application in new domains, or improvements in computational efficiency in Section 8 *“Conclusion*”.

**Comment#18:** Apply BPFCGs to a specific industry problem (e.g., market competition) and analyze their effectiveness compared to traditional models.

**Response.** We highlighted the application of BPFCGs in the market competition in telephonic companies in section*, “*Analysis of competition among di*ff*erent cell phone companies through BPFCGs,” and analyzed the effectiveness of the application of BPFCGs compared to traditional models in section 5.1, *“*Prevalence of our presented model for mobile companies through BPFCGs.”

**Comment#19:** Present a table comparing BPFCGs with existing models in terms of advantages, limitations, and key features.

**Response.** We have presented a table comparing BPFCGs with existing models in terms of advantages, limitations, and key features in Section 5.1, *“Prevalence* of our presented model for mobile companies through BPFCGs (Tables. 19, 20 & 21)”.

**Comment#20:** Ensure grammatical accuracy, proper citation formatting, and adherence to journal guidelines.

**Response.** As per reviewer’s suggestion, we have ensured grammatical accuracy, proper citation formatting, and adherence to journal guidelines.
