# Supplementary material for: A study of competitions in different fields through graphs under bipolar picture fuzzy environment
Source: PLoS One. 2025 Oct 10;20(10):e0329192. doi: 10.1371/journal.pone.0329192 (PMC12513671; doi:10.1371/journal.pone.0329192)
Supplement: Attachment — Submitted filename: Reviewers comments with Responses 2.docx [file pone.0329192.s002.docx]

**Manuscript ID:** PONE-D-25-05783

**Title:** A study of competitions in different fields through graphs under bipolar picture fuzzy environment

We sincerely appreciate the reviewer for their time and valuable comments. There is no doubt that incorporating these suggestions has enhanced the overall quality of our article. A point-by-point response to the reviewer’s comments is provided below.

**Comment#1:**  Check all the citations, for example: Cuong [27] should be Cuong and Kreinovich [27]
**Response.** Thank you very much for your suggestion. We have corrected and replaced Cuong by Cuong and Kreinovich. Moreover, we have revised citations and corrected wherever we have found such mistakes.

**Comment#2:** Improve the quality of all figures, for example Figure 1,7,....”

**Response.** As per reviewer suggestion, we have improved the quality of almost all the figures.

**Comment#3:**  Improve the presentation follow the format of PLOS One.

**Response.** As per reviewer’s suggestion, we have followed the format of PLOS ONE, properly.

**Comment:** Check and improve the quality of English.

**Response.** Following the reviewers’ suggestions, we have revised the manuscript and improved the quality of English—from the abstract to the conclusion—wherever necessary.

**Comment:** Page 38,39 N. Deva should be Deva.

**Response.** As per the reviewer’s suggestion, we have replaced “N. Deva” with “Deva.” Moreover, we have made similar corrections wherever applicable throughout the manuscript.
